# Nanoparticle-based drug delivery systems targeting cancer cell surfaces

**DOI:** 10.1039/d3ra02969g

**Published:** 2023-07-17

**Authors:** Liquan Hong, Wen Li, Yang Li, Shouchun Yin

**Affiliations:** a Deqing Hospital of Hangzhou Normal University, The Third People's Hospital of Deqing Deqing 313200 China yinsc@hznu.edu.cn; b College of Material, Chemistry and Chemical Engineering, Hangzhou Normal University, Key Laboratory of Organosilicon Chemistry and Material Technology, Ministry of Education, Key Laboratory of Organosilicon Material Technology Zhejiang Province Hangzhou 311121 China

## Abstract

Traditional cancer chemotherapy easily produces serious toxic and side effects due to the lack of specific selection of tumor cells, which restricts its curative effect. Targeted delivery can increase the concentration of drugs in the target site and reduce their toxic and side effects on normal tissues and cells. Biocompatible and surface-modifiable nanocarriers are novel drug delivery systems, which are used to specifically target tumor sites in a controllable way. One of the effective ways to design effective targeting nanocarriers is to decorate with functional ligands, which can bind to specific receptors overexpressed on the surfaces of cancer cells. Various functional ligands, including transferrin, folic acid, polypeptide and hyaluronic acid, have been widely explored to develop tumor-selective drug delivery systems. This review focuses on the research progress of various receptors overexpressed on the surfaces of cancer cells and different nano-delivery systems of anticancer drugs targeted on the surfaces of cancer cells. We believe that through continuous research and development, actively targeted cancer nano-drugs will make a breakthrough and become an indispensable platform for accurate cancer treatment.

## Abstract 2

This review focuses on the research progress of various receptors overexpressed on the surface of cancer cells and different nano-delivery systems of anticancer drugs targeted on the surface of cancer cells.

## Graphical abstract

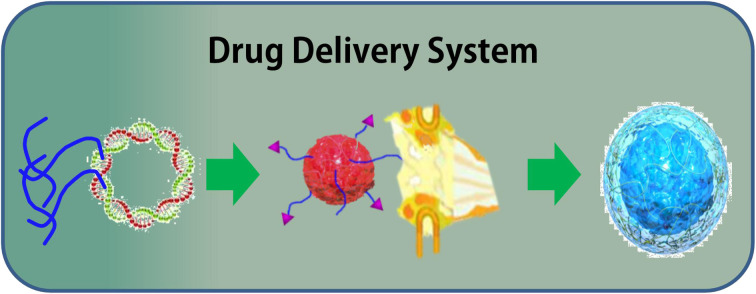

## 1. Introduction

Malignant tumors are a common public health problem faced by human beings all over the world, which seriously endanger the life, health and quality of life of patients. According to the latest statistics of the World Health Organization (WHO), it is estimated that 19.3 million new cancer cases and 10 million cancer deaths occurred worldwide in 2020.^1,2^ Traditional cancer treatment methods include surgery, chemotherapy and radiotherapy.^3,4^ Chemotherapy is the most commonly used cancer treatment strategy and can also be used in combination with other methods. Although these conventional methods have achieved clinical success to some extent, many chemotherapy drugs will lead to serious side effects because of their short half-life and lack of targeting ability.^5,6^ In addition, tumor cells will also be resistant to drugs, which limits the clinical application of some chemotherapy drugs.^7^ Therefore, it is an urgent and important problem to explore and develop more effective methods to selectively deliver drugs to tumor sites. With the development of nanotechnology, nano-medicine has shown a good application prospect in improving cancer treatment.^8^ Compared with individual drug delivery, nano-delivery system can prolong drug half-life to reduce side effects, and improve drug accumulation in tumor through passive or active targeting, which has greater advantages in cancer treatment.^8–10^ It is crucial to develop effective nano-drug carriers to deliver drugs in nano-delivery system. Nano-drug delivery carrier, as a nano-sized drug carrier system, can improve the safety of chemotherapy drugs used for antitumor and improve the therapeutic effect.^7,11^ Common nano-drug carriers mainly include polymer nanocarriers, liposomes and inorganic nanocarriers (such as gold nanocarriers, silica nanocarriers and magnetic nanocarriers, *etc.*)^12–15^

**Fig. 1:**
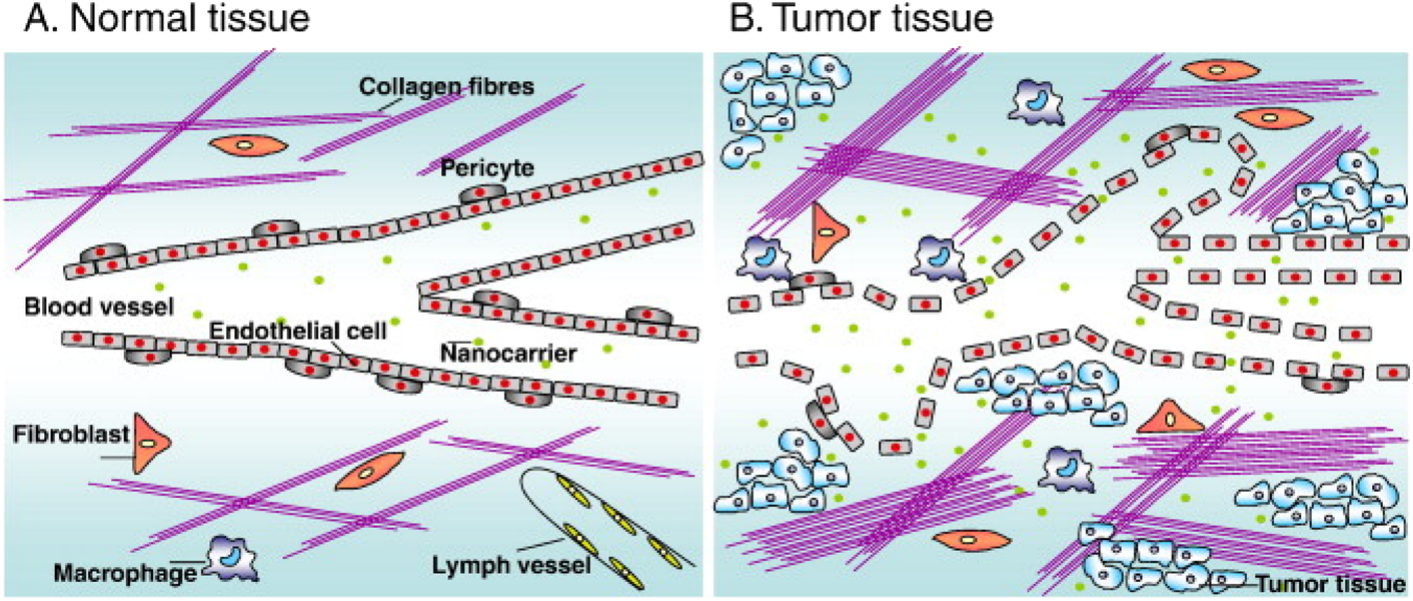
(A) Normal tissues maintain vascular integrity by peripheral cells and lymphatic vessels exist. (B) The tumor tissue contains defective blood vessels, many cystic structures and perforations, and lymphatic vessels are lacking. Reprinted with permission from ref. 16. Copyright @2010 Elsevier.

Compared with normal tissues, tumor tissues have abundant blood vessels, irregular blood vessel wall cells, and nanoparticles are easy to ooze from tumor blood vessels (Fig. 1). In addition, the increase in the number of collagen fibers, fibroblasts, and macrophages in tumor tissue, as well as the dysfunction of the lymphatic drainage system, lead to the inability to effectively remove nanocarriers entering the tumor and remain in the tumor for a long time. This passive phenomenon is called “enhanced permeability and retention (EPR) effect” of tumor.^16–19^ The exudation strength of nanoparticles depends on the gap size of endothelial cells and the size of cross-endothelial channels.^20,21^ Generally, the smaller the particles, the easier it is to reach the tumor site through EPR effect.^22^ However, the nanoparticles should not be too small, otherwise they will be easily removed by the kidney or invaded into capillaries, and at the same time, they should not be too large to escape the phagocytosis of reticuloendothelial system (RES) and the clearance of immune system.^23,24^ Considering all these factors, the effective nanocarriers should be in the diameter range of 10–150 nm.^25^ Although EPR effect alleviates the dilemma that chemotherapy drugs are easy to be eliminated *in vivo* and have no targeting ability, it is difficult to obtain high enough drug concentration in tumor tissue through passive targeting, so the curative effect is not significant.

Due to the defects of passive targeting, it is necessary to explore the active targeting strategy of nanocarriers.^26,27^ Active targeting strategy mainly depends on the difference of pathophysiology and microenvironment between tumor site and normal tissue. Compared with normal cells, many receptors on the surface of cancer cells or tumor vascular endothelial cells are overexpressed, such as folate receptor (FR), transferrin receptor (TfR), epidermal growth factor receptor (EGFR) and integrin receptor (shown in Table 1).^28–30^ It is the overexpression of these special receptors that can couple different types of ligands, such as antibodies, peptides, protein, nucleic acids and polysaccharides, to the surface of nanocarriers, so as to increase the specific recognition between the carriers and cancer cells.^23^ When the nanocarriers bind to the cell surface receptors, they internalize into cancer cells through receptor-mediated endocytosis to maximize drug accumulation.^31^ The delivery ability of nanoparticles is directly related to the structure and composition of nanoparticles. The main challenge of developing actively targeted nanoparticles is that the required nanoparticles must be able to reach the vicinity of the target and interact with it. Therefore, based on the overexpression of specific receptors on tumor cells, the development of active targeting nano-drugs targeting the surface of cancer cells has attracted great attention of researchers. In this review, some receptors overexpressed on the surface of cancer cells are introduced, and the research progress of anti-cancer drugs delivery through targeted receptors on the surface of cancer cells actively targeting nanomaterials is reviewed.

**Table 1:** Various receptors overexpressed on different cancer cells along with their targeting moieties

Receptor	Targeting moieties	Cancer type
Transferrin	Transferrin receptor ligand	Pancreatic cancer, colon cancer, bladder cancer
	Transferrin	
Folate	Folic acid	Ovarian cancer, lung cancer, breast cancer, cervical cancer, renal cancer, brain cancer
EGFR	Anti-EGFR	Colon cancer, breast cancer, head, neck cancer, ovarian cancer
CD 44	Hyaluronic acid	Liver cancer, breast cancer, colon cancer, lymphoma
Integrin	RGD peptide	Glioma, melanoma, lung cancer, breast cancer

## 2. Cell membrane surface receptor

### 2.1. Transferrin receptor

Transferrin (Tf) is a transmembrane glycoprotein, which is used to transport and regulate the distribution of iron in human cells.^32,33^ It binds to transferrin receptor (TfR) and mediates the intracellular transport of iron, which is an important way for cells to obtain iron. Usually, Tf combines with iron in serum to transport iron to liver and other cells, and then combines with TfR on the cell surface to form Tf–TfR complex, which is then absorbed into cells through internalization.^34^ Due to iron being an essential protein cofactor and participating in basic life processes such as cell proliferation and growth, many tumor cells have stable and highly expressed TfR on their surface.^35^ The expression of transferrin receptor in common tumors such as pancreatic cancer, colon cancer and bladder cancer are up-regulated, which is higher than that in normal cells.^33^ Moreover, the expression of TfR is related to the malignant level of tumor, and TfR is more expressed in breast cancer, glioma, lung adenocarcinoma, chronic lymphocytic leukemia and liver cancer with high malignant degree and easy metastasis.^36^ This has attracted the attention of researchers, making it an active targeting strategy to deliver drugs to tumor cells. TfR can be used either to target the delivery of drugs into cancerous cells or to prevent the normal function of the receptor leading to cancer cell death. Up to now, transferrin receptor has become the most widely studied cell target in anticancer.

### 2.2. Folate receptor

**Fig. 2:**
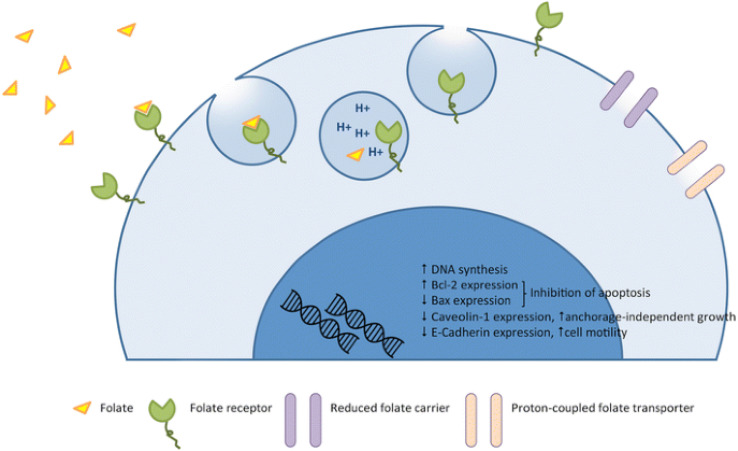
Mechanism of action of folate and the folate receptor against the tumor. Reprinted with permission from ref. 45. Copyright @2015 Spring Nature.

Folic acid (FA) also known as vitamin B9, plays an essential role in DNA synthesis and replication, cell division, growth and survival.^37^ Folic acid is usually combined with folate receptor (FR) and transported across the membrane through endocytosis. As shown in Fig. 2, after FA binds to FR, it forms vesicles and enters cells through endocytosis.^38^ After the fusion of intracellular vesicles and lysosomes, acidification leads to the release of FA into cells, and FR is transferred to the surface of cell membrane again.^39,40^ FR, also known as folic acid binding protein, is a glycosyl phosphatidylinositol ankyrin with four subtypes: FRα, FRβ, FRγ and FRδ.^41^ FRα, FRβ and FRδ are membrane proteins fixed by glycosyl phosphatidylinositol,^42^ while FRγ is a secretory protein of lymphoid cells.^43^ Among them, FRα has a high affinity for FA.^44^ The expression level of FRα in normal tissues is very low,^45^ and most of FRα is expressed at the apical (luminal) surface of epithelial cells, which will not directly interact with FA or molecules targeted by FR in the circulatory system.^46,47^ However, FR is highly expressed in some epithelial tumors, including ovarian cancer, lung cancer, breast cancer, endometrial cancer, cervical cancer, renal cancer, bladder cancer and brain cancer,^48^ in order to meet high folate demand of rapidly dividing cells.^49^ Cancer cells can make FRα approach FA by changing its transmembrane region, transcription level and translation level,^50^ and can also promote the growth and proliferation of tumor cells through signal transduction and transcription factors.^45^ Therefore, FRα has become an attractive therapeutic receptor for specific drug delivery.

### 2.3. Epidermal growth factor receptor

**Fig. 3:**
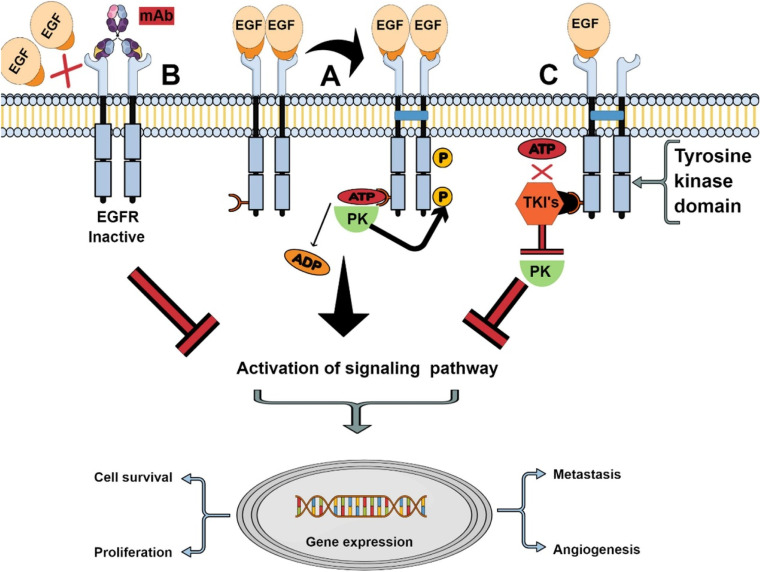
The mechanism of tyrosine kinase inhibitors and anti-EGFR monoclonal antibodies. Reprinted with permission from ref. 61. Copyright @2021 Elsevier.

Epidermal growth factor receptor (EGFR), also called HER1 (human epidermal receptor 1) or ErbB1 (receptor tyrosine-protein kinase erbB-1), is a transmembrane glycoprotein belonging to tyrosine kinase receptor family.^51^ The EGFR family also includes three other family members with similar structures: HER2, HER3 and HER4, all of which contain the extracellular ligand-binding domain, the intracellular tyrosine kinase domain (except HER3) and the intermediate transmembrane structure.^52^ EGFR is distributed on various cell surfaces and plays an important role in cell proliferation, differentiation and other life activities.^53^ When endogenous ligands such as epidermal growth factor (EGF) bind to EGFR, kinases will dimerize,^54^ γ-phosphate will be transferred from adenosine triphosphate (ATP) to kinase domain,^55^ and phosphorylated EGFR can activate downstream signal pathways (such as MAPK and P13K pathways),^51^ thus regulating cell proliferation and differentiation and other life activities. Overexpression or mutation of EGFR family will lead to kinase dysfunction, which will lead to abnormal cell life activities and eventually tumor. Therefore, EGFR is a key participant in the development of many cancers (such as colon cancer, non-small cell lung cancer, breast cancer, head and neck cancer and ovarian cancer)^56^ and has become an important drug target.^57^ At present, targeted drugs for EGFR mainly include small tyrosine kinase inhibitors (TKIs) and monoclonal antibodies (mAb), which have been developed and successfully used in clinic.^52^ The mechanism of tyrosine kinase inhibitors and anti-EGFR monoclonal antibodies in cancer cells is shown in Fig. 3. TKIs inhibits tyrosine kinase phosphorylation by interacting with intracellular domains, thus inhibiting the activation of EGFR signaling pathway.^58^ Common TKIs include the first generation gefitinib, erlotinib, the second generation afatinib, dacomitinib and the third generation osimertinib.^59^ However, mAb specifically binds to the extracellular domain of EGFR, blocking the binding of ligands, thus cutting off the EGFR signaling pathway.^60^ Cetuximab (Erbitux) and panitumumab (Vectibix) are both monoclonal antibodies approved by FDA.^61^ Therefore, EGFR is a mature and attractive receptor for targeted therapy of specific drugs on the surface of cancer cells.

### 2.4. Cluster of differentiation 44

Cluster of differentiation 44 (CD44) is a single-chain transmembrane adhesion glycoprotein, which is involved in many signal pathways in physiological and pathological processes, especially the occurrence, progress and metastasis of tumors.^62^ CD44 is overexpressed on the surface of many tumors, including liver cancer, breast cancer, colon cancer, lymphoma, *etc.*^63^ It is considered as a marker of many cancer stem cells (CSCs),^64^ so it can be a cell surface target for active drug targeting and treatment. CD44 can interact with various cytokines, growth factors and extracellular matrix (ECM) secreted by cells in tumor microenvironment.^65^ The common ligands of CD44 include hyaluronic acid (HA), osteopontin (OPN), chondroitin and so on.^66^ Among them, HA is the main component of extracellular matrix,^67^ and its binding with CD44 is cell-specific. In addition, HA is a natural polymer which is easy to biodegrade and has good biocompatibility. Usually, HA-modified nanocarriers are used to specifically bind CD44, and at the same time, the binding between endogenous HA and CD44 is disturbed, which weakens the intracellular signaling pathway and eventually leads to cell apoptosis.^68^

### 2.5. Integrin receptor

Integrin is a transmembrane glycoprotein receptor, which is expressed on various cell surfaces. It is involved in a variety of cell signal transduction pathways and is closely related to the proliferation, invasion and migration of cancer cells.^69^ Integrin is composed of an α subunit and a β subunit through non-covalent bond to form a heterodimer structure.^70^ Up to now, it has been found that there are 18 α subunits and 8 β subunits, which together form 24 integrin receptors.^71^ Each integrin can recognize and bind to ECM ligands, cell surface ligands or soluble protein ligands, thus widely regulating cell functions.^72^ Arginine–glycine–aspartic acid (RGD) sequence in ECM is a common binding motif of integrins, and it has the strongest binding ability with α_ν_β_3_ and α_ν_β_5_ integrins that are not expressed in normal tissues.^73^ α_ν_β_3_ integrin is overexpressed in tumor blood vessels and can specifically recognize RGD on ECM. Therefore, integrin has become one of the important targets of drug development.

## 3. Nanoparticles targeting cancer cells

Next, some nanoparticle-based targeted formulations that have shown positive results in recent studies are presented (Table 2).

**Table 2:** Some formulations of nanoparticles with positive results in the recent investigations

Nanoparticle	Anticancer drug	Targeting agent	Reference
Pluronic P123	Paclitaxel (PTX)	Transferrin	74
PEG	4-Methylumbelliferone	Folic acid, HA	75
DSPE–PEG	Doxorubicin (DOX)	RGD peptide	76
DSPE–PEG	Doxycycline, docetaxel	Folate receptor beta (FRβ)	77
Poly(amidoamine) dendrimers	Doxorubicin (DOX)	EGFR-binding peptide 1	78
Gold nanorods	Paclitaxel (PTX), curcumin (CUR)	cRGD peptide	79
Gold nanoparticle	p53DNA	Antibody EGFR (C225)	80
Gold-polyvinylpyrrolidone	Curcumin (CUR)	Folic acid	81
Chitosan-coated mesoporous silica	Gemcitabine	Transferrin	82
Chitosan-coated mesoporous silica	Doxorubicin (DOX)	EGFR/HER2 aptamer	83
Superparamagnetic iron oxides (SPIO)	Doxorubicin (DOX)	Folic acid	84

### 3.1. Polymer delivery system

In the past few decades, polymer delivery system has been widely explored as a drug delivery system for biomedical applications. Polymer micelles synthesized from the polymer materials have significant applications in cancer diagnosis and treatment because of its unique advantages. Polymer micelles is usually a “core–shell” structure formed by self-assembly of amphiphilic block copolymer in aqueous solution. Its hydrophobic core can carry hydrophobic drugs, thus improving the solubility and stability of drugs; hydrophilic shell forms a protective barrier by hydration, which reduces the absorption of protein and the recognition and clearance of reticuloendothelial system (RES) during blood circulation, and prolongs the half-life of drugs.^85^ The hydrophilic shell of polymer is easy to be chemically modified to achieve active targeting. In particular, polymer-based nano micelles such as polyethylene glycol (PEG), polylactic acid (PLGA) and chitosan (CS) modified by cancer cell surface-related ligands have been designed to treat breast cancer, glioma, prostate cancer, lung cancer and so on.

**Fig. 4:**
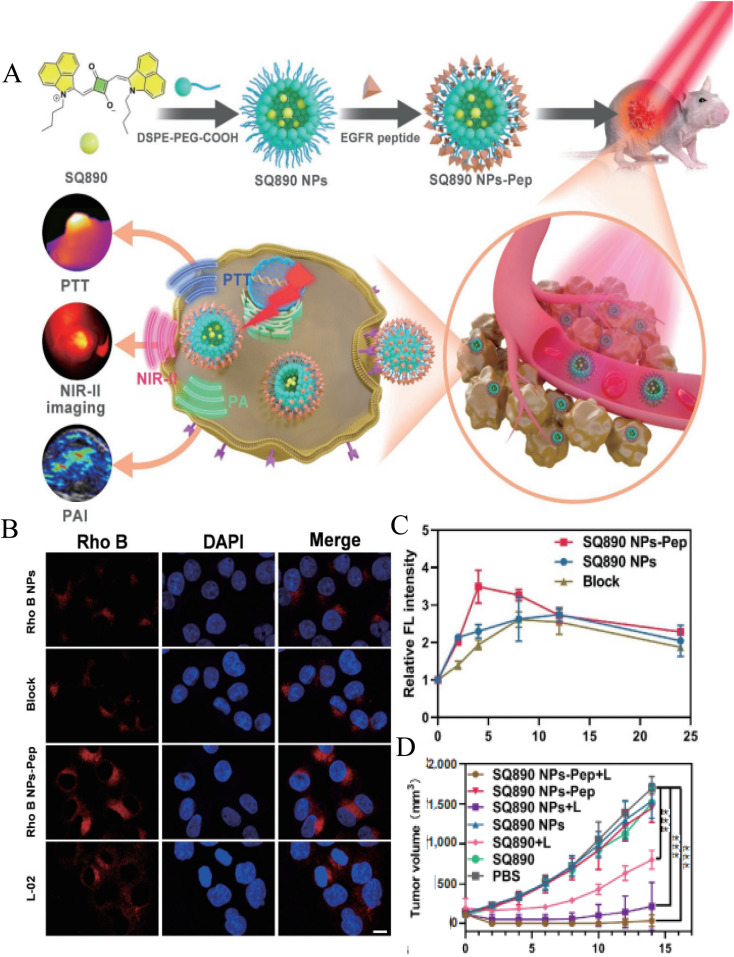
(A) Schematic illustration of the EGFR-targeting nanoparticle SQ90 NPs-Pep for photoacoustic NIR-II fluorescence dual-modality imaging-guided PTT of oral cancer; (B) cellular uptake (CAL 27 and L-02 cells) of Rho B-labeled NPs observed by CSLM; (C) semiquantitative analysis of relative fluorescent intensity at different time points after various treatments; (D) tumor volume growth of mice with CAL 27 tumors in each group. Reprinted with permission from ref. 87. Copyright @2022 Springer Nature.

Ge *et al.* co-encapsulated paclitaxel (PTX) and photosensitizer (5-ALA) into nanoparticles (Tf-5-AlA-PTX-NCs) with magnetic carrier modified by Tf-coupled copolymer Pluronic P123, as a new strategy of combined chemotherapy and photodynamic therapy.^74^ They proved that Tf-5-ALA-PTX-NCs modified with transferrin ligand showed stronger fluorescence signal in MCF-7 cell xenotransplantation tumor compared with 5-ALA-PTX-NCs treatment, thus enhancing the antitumor effect. Hu *et al.* developed a hyaluronic acid–cysteamine–polylactic acid–glycolic acid (HA–SS–PLGA) polymer nano-delivery system to deliver doxorubicin (DOX) and cyclopamine to breast cancer cells with CD44 overexpression.^67^ They found that the intake of PLGA–DOX and HA–SS–PLGA–DOX into MCF-7 cells was similar. However, HA–SS–PLGA–DOX particles are easier to enter MDA-MB-231 cells with CD44^+^ overexpression than PLGA–DOX, and MDA-MB-231 is more cytotoxic to MDA-MB-231. They proved that the modification of HA can enhance the targeting ability of nano-drug delivery system to CD44 overexpressed cancer cells. Pan *et al.* constructed two polymer micelles based on pentaerythritol polycaprolactone-*b*-poly(*N*-isopropylacrylamide) and pentaerythritol polycaprolactone-*b*-poly(*N*-vinylcaprolactam) to deliver DOX for targeted treatment of glioma.^86^ The cellular uptake study showed that FA receptor promoted the accumulation of folate-coupled polymer micelles in C6 glioma cell lines. In addition, compared with normal cells (HaCaT), the nano-system has a selective killing effect on cancer cells, thus reducing the side effects of drug-loaded carriers. Recently, Ling *et al.* used DSPE–PEG5K–COOH to wrap NIR-II dye SQ890 to form nanoparticles, and modified EGFR targeting peptide (GE890) on its surface, which can accurately target to the tumor site, so as to realize photoacoustic (FA)/NIR-II fluorescence dual-mode imaging to guide PTT to treat oral cancer (Fig. 4A).^87^ As shown in Fig. 4B, compared with L-02 cells with low EGFR expression, the nanoparticles modified by targeted peptides have more uptake in CAL 27 cells with high EGFR expression. Finally, SQ890 NPs-Pep showed better imaging ability of NIR PA/NIR-II fluorescence imaging and higher PTT effect in mice (Fig. 4C and D). All these results reveal the EGFR targeting effect of cetuximab against immune micelles. Amit S. Yadav *et al.* developed an RGD peptide functionalized chitosan nano-delivery system (RGD-CHNPs) to deliver raloxifene (Rlx).^88^ Compared with non-targeting CHNPs, RGD-CHNPs showed higher tumor accumulation. Critically, Rlx–RGD-CHNPs have no toxicity to healthy tissues, but show significant antitumor effect on breast tumors expressing α_v_β_3_ integrin due to the active targeting of RGD peptide. Marwa Labib Essa *et al.* prepared CD44 and folate receptor dual-targeted nanoparticles (4-MU@FA–PEG–HA NPs) based on folic acid–polyethylene glycol–hyaluronic (FA–PEG–HA) to improve the therapeutic efficacy of 4-methylumbelliferone (4-MU) on lung cancer.^75^ The cytotoxicity analysis showed that the IC_50_ of 4-MU was reduced from 224.5 to 64.75 μg ml^−1^ in the case of 4-MU@FA–PEG–HA NPs.

Due to their good biocompatibility and biodegradability, many polymer materials have been used to develop nano-drug delivery systems for targeted delivery of cancer cells. However, there are still some problems in its clinical application and transformation potential, such as poor micelle stability and premature drug release. It is still urgent to develop a stable, multifunctional and on-demand intelligent polymer delivery system.

### 3.2. Liposome delivery system

Liposome delivery systems are the most commonly used research nanocarriers for tumor targeted drug delivery. In the 1960s, Bangham first reported the structure with liposomes.^89^ Liposomes are tiny vesicles composed of natural phospholipids and cholesterol, including a phospholipid bilayer and a hydrophilic inner compartment. Generally, hydrophilic drugs are wrapped in hydrophilic inner compartments, while hydrophobic drugs are embedded in hydrophobic double-layer membranes.^90^ This unique encapsulation ability makes it have the advantages of good biocompatibility, high drug loading rate, no immunogenicity and easy surface modification.^76^ In 1995, PEGylated liposome (Doxil) coated with anticancer drug DOX was approved by FDA for clinical cancer treatment, and became the first nano-drug on the market.^91^ Since then, many liposome-based nano-drugs have been researched and developed, and many liposome drugs such as DepoCyt, DepoDur, Exparel, Marqibo and DaunoXome have been approved for clinical research.^92,93^ Liposome is the most successful nano-drug delivery carrier in clinical application so far. PEG modification can endow liposomes with “stealth” function and prolong the circulation time *in vivo*.^94^ However, only relying on the EPR effect of liposomes, the enrichment rate of drugs in tumor sites is still low. In order to achieve selective delivery, many drugs with different structures can be modified on the surface of liposomes, such as some targeting ligands, to target the specific receptors of tumor cells, thus improving the therapeutic effect.

**Fig. 5:**
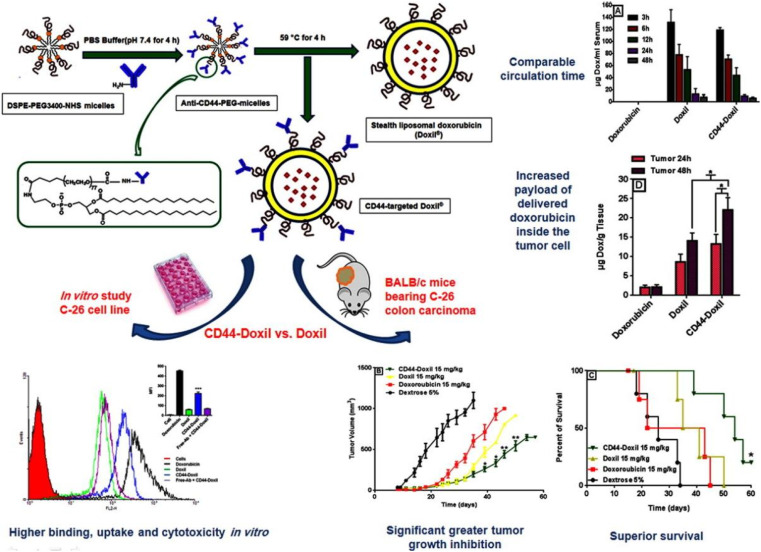
Preparation method of CD44-Doxil and therapeutic effect *in vivo* and *in vitro*. Reprinted with permission from ref. 95. Copyright @2015 Elsevier.

Arabi *et al.* functionalized Doxil with CD44 monoclonal antibody (mAb) and compared its antitumor activity with Doxil.^95^*In vitro* results showed that the uptake of CD44-targeting (mAb)-modified Doxi in CD44 mouse colon cancer cells was significantly improved compared with Doxil (Fig. 5). Furthermore, CD44-Doxil exhibited higher doxorubicin concentrations within tumor cells as well as excellent antitumor efficacy and improved therapeutic effect compared with Doxil-treated C-26 colon cancer mice. Amin *et al.* designed liposomes containing doxorubicin modified with two ligands: RGD and type 1 transactivator of transcription (TAT) peptides.^76^ They found that the presence of two active ligands together increased the targeting area and improved the binding of liposomes to cells, showing enhanced therapeutic effect on the B16 tumor model. Yong Il Park *et al.* constructed a FRβ-targeted pH-sensitive liposome (FRβ-pH lipo) to deliver doxycycline and docetaxel to better inhibit tumor growth in non-small cell lung cancer (NSCLC).^77^ It was observed by laser confocal that in FRβ overexpressed A549 cells, the fluorescence signal of folate-modified FRβ-pH lipo-Cy5.5 was 7.23 times stronger than that of NH_2_-pH lipo-Cy5.5. Song *et al.* prepared biodegradable liposome nanoparticles (LPN) coupled with epidermal growth factor receptor to co-deliver docetaxel (DTX) and resveratrol (RSV) for the treatment of advanced non-small cell lung cancer.^96^ Sonali *et al.* studied a therapeutic diagnostic liposome conjugated with transferrin (DTX-QD-TPGS-Tf) for co-delivery of docetaxel (DTX) and quantum dots (QD) to brain cancer cells.^97^ Compared with Docel, the liposome modified by transferrin receptor shows significantly higher delivery of DTX and QD to brain cancer sites. After 4 hours, the drug accumulation efficiency of targeted liposomes was 10.56 times that of Docel.

Generally, the structure of liposome endows it with unique characteristics and functions, making liposome an ideal carrier for various therapeutic drugs and clinical applications. Liposomes modified by targeting ligands increase the target accumulation of drugs and greatly reduce the off-target toxicity of various drugs. However, the complexity of functionalized liposomes increases, which will hinder the transfer to large-scale production and clinic. Therefore, the development of functional liposomes from the bench to the bedside is still the focus of researchers.

### 3.3. Dendrimer nanocarriers

Dendrimers are highly branched macromolecules assembled from monomeric structures through covalent bonds. Its 3D branched structure with multiple functional groups on the surface makes it multifunctional and biocompatible.^98^ Due to its good water solubility, biodegradability, low polydispersity, controllable molecular size, and high adaptability of surface chemistry, it has become an excellent carrier candidate for biological and drug delivery systems.^99^ Among various nanocarriers, dendrimers have received great attention from researchers. Therefore, dendrimers can be used as a very promising drug delivery system, which is expected to achieve drug targeting to specific cells. Gong *et al.* designed a hydrophilic dendritic copolymer as a platinum-based drug nanocarrier targeting ovarian cancer, composed of poly(amidoamine)-*b*-poly(aspartic acid)-*b*-poly(ethylene glycol) conjugated to cRGD peptide and anthocyanin 5 (Cy5) fluorescent dye.^100^ Cell uptake experiments demonstrated that cRGD conjugation can effectively enhance the uptake of nanoparticles in cancer cells through integrin-mediated endocytosis. Importantly, carboplatin-complexed cRGD-conjugated nanoparticles were more cytotoxic to ovarian cancer cells than non-targeting nanoparticles.

**Fig. 6:**
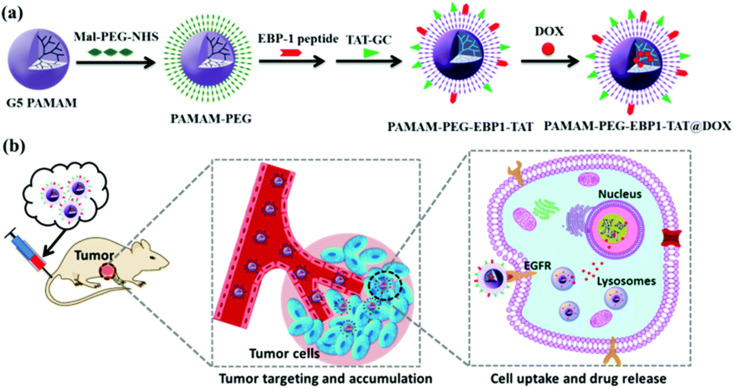
(a) Construction of the dual-functional dendrimer-based drug carrier and (b) schematic illustration of tumor targeting therapy using the dual-functional drug delivery system. Reprinted with permission from ref. 78. Copyright @2019 The Royal Society of Chemistry.

Poly(amidoamine) (PAMAM) dendrimers have high-density amino groups on the surface, which can be easily modified and functionalized by chemical coupling with various functional molecules.^101^ PAMAM dendrimers have been developed to the fifth generation (G5) at present, and are uniform spherical shapes with small size.^102^ Hong *et al.* synthesized acetamide-terminated G5 dendrimers (G5-Ac-AF488-FAx) functionalized with 2–14 folic acid molecules and Alexa Fluor 488 dye (AF488).^103^ They found that the binding affinity of G5-Ac-AF488-FAx with folate-binding protein (FBP) was ∼2500 to ∼170 000-fold stronger than free FA and increased the residence time of the nanocomplexes on cells. Wang *et al.* covalently coupled fluorescein isothiocyanate (FI) and FA to G5 PAMAM dendrimers (G5.NH_2_) with acetyl terminal groups, and the anticancer drug 2-methoxyestradiol (2-ME) targeted delivery to cancer cells overexpressing FAR.^102^ The G5.NHAc–FI–FA/2-ME complex could specifically target human epithelial carcinoma (KB) cells with high levels of FAR and showed more toxicity. Liu *et al.* established a bifunctional drug delivery system based on PAMAM dendrimers coupled with EGFR-binding peptide 1 (EBP-1) and cell-penetrating peptide trans-activating transcriptional activator (TAT), using for encapsulating doxorubicin (DOX) (Fig. 6).^78^ This bifunctional dendritic polymer drug carrier significantly improves the drug accumulation at the tumor site, improves the inhibitory effect of DOX on human breast cancer, and reduces the systemic toxicity of DOX *in vivo*.

In a word, targeted nano-delivery systems based on dendrimers have been widely studied because of their promising characteristics. However, due to the complexity of multi-component 3D structure, careful design is needed to ensure repeatable formula in mass manufacturing process.

### 3.4. Inorganic nanocarriers for drug delivery

Inorganic delivery systems are widely used in biomedicine and other fields because of their diverse structures and functions, such as adjustable size, shape, high specific surface area, unique optics and magnetism, and easy surface modification. The most important thing is that it is easy to modify, and different ligands can be used to functionalize nanocarriers, thus improving their attractiveness to targets. Therefore, inorganic nanocarriers are excellent prospects in targeted drug delivery, imaging diagnosis and drug synergistic therapy. The main inorganic nanocarriers include: gold nanocarriers, mesoporous silica nanocarriers, magnetic nanocarriers, *etc.*

#### 3.4.1. Gold nanocarriers

Gold nanocarriers are widely used in the biological field due to their unique size, controlled release of drugs, and lower toxic side effects than other metals. The surface of gold nanocarriers is easy to modify, and some organic compounds (*e.g.*, DNA and peptides) can be functionalized through Au–S bonds or Au–thiol bonds, which greatly improves its bioavailability,^104^ making it useful in tumor imaging, diagnosis and treatment.

**Fig. 7:**
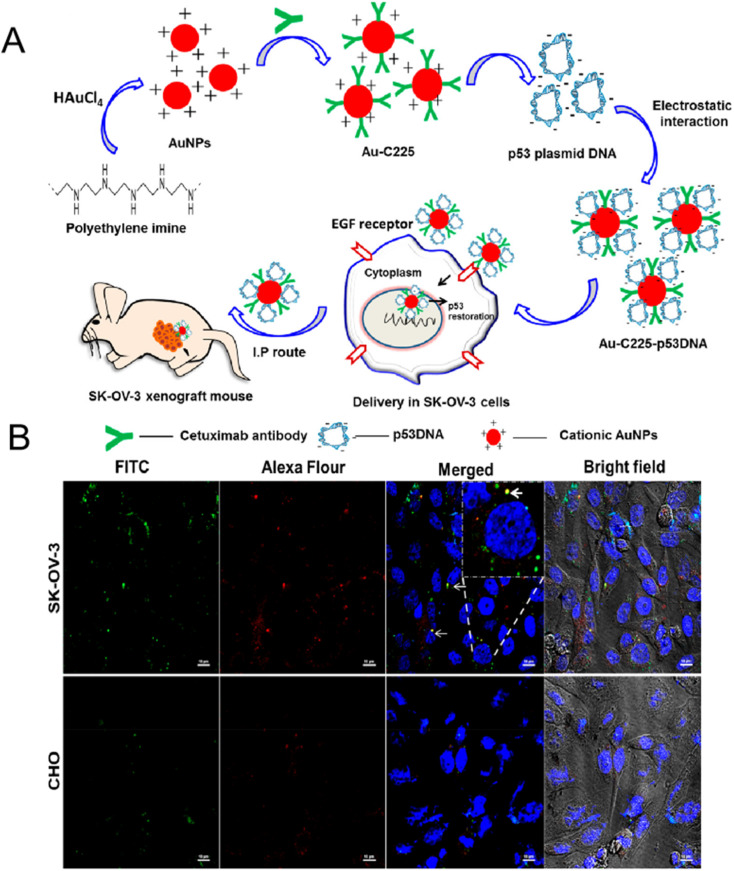
(A) Preparation and mechanism diagram of Au-C225-p53DNA; (B) confocal uptake of Au-C225-p53DNA in SK-OV-3 cells (top) and CHO cells (bottom). Reprinted with permission from ref. 80. Copyright @2019 American Chemical Society.

Kim *et al.* synthesized a radioactive iodine-labeled, cyclic RGD-PEGylated gold nanoparticle probes (^125^I-cRGD-AuNP) for tumor site imaging.^104^ Experiments showed that the binding affinity of ^125^I-cRGD-AuNP probes to α_v_β_3_ integrin was 150-fold higher than that of unmodified free cRGD. *In vivo* single-photon emission computed tomography (SPECT/CT) imaging results showed that the ^125^I-cRGD-AuNP probes could rapidly and efficiently target α_v_β_3_ integrin overexpressing tumor sites within 10 minutes after injection. Due to the unique optical properties of gold, functionalized gold nanocarriers are not only used for *in vivo* imaging, but also used in the targeted therapy of tumors. Zhu *et al.* utilized gold nanorods, cRGD peptide, paclitaxel (PTX), and curcumin (CUR) to construct tumor-targeting and multi-stimuli-responsive nanocarriers (PTX/CUR/Au NRs@cRGD) for chemo-photothermal synergistic therapy.^79^ Through the analysis of *in vivo* behavior, DiR/PEG/Au NRs did not observe fluorescence signal in A549 tumor, while DiR/PEG/Au NRs@cRGD showed obvious accumulation of fluorescence signal in the tumor site, and presented an ideal tumor ablation effect. Rajesh Kotcherlakota *et al.* designed a cationic gold nanoparticle functionalized with monoclonal antibody EGFR (C225) and wild type p53 plasmid DNA (p53DNA) for targeted gene delivery to ovarian cancer (Fig. 7A).^80^ The intracellular uptake of Au-C225-p53DNA was observed in SKOV-3 cells (human ovarian cancer) and CHO cells (china hamster ovary), respectively. As shown in Fig. 7B, the fluorescence intensity of Au-C225-p53DNA in EGFR overexpressing SKOV-3 cells was much more obvious than that in CHO cells (Fig. 7B). In nude mice bearing SKOV-3 xenotransplantation model, Au-C225-p53DNA treatment group showed effective tumor targeting and significant tumor regression. Sneha Mahalunkar *et al.* designed gold–polyvinylpyrrolidone nanoparticles (FA–CurAu–PVP NPs) modified with folic acid and curcumin for targeted delivery in a breast cancer model system.^81^*In vitro* experiments demonstrated that folic acid-modified FA–CurAu–PVP NPs had a higher potential in inhibiting the proliferation and migration of breast cancer cells. FA–CurAu–PVP had higher anticancer activity in triple-negative breast cancer cells with higher folate receptor expression than other breast cancer cells. Liu *et al.* employed CD44-specific targeting of hyaluronic acid (HA) and cationic bovine serum albumin-protected gold nanoclusters to construct a size-reducible nanoplatform (AuNC@CBSA@HA).^105^ AuNC@CBSA@HA was further loaded with paclitaxel (PTX) and indocyanine green (ICG) for chemical photothermal therapy. Among them, HA not only endows nanoparticles with active targeting properties, but also degrades at the tumor site to reduce the size of nanoparticles and enhance the penetration depth of drugs in tumors. The Au nanoclusters finally inhibited 95.3% of orthotopic tumor growth and 88.4% of lung metastatic growth in 4T1 tumor-bearing mice.

**Fig. 8:**
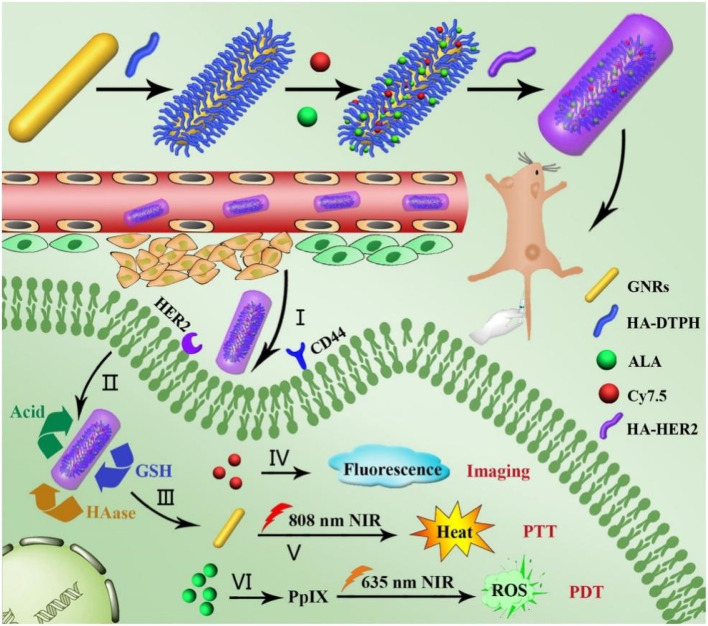
Schematic diagram of the preparation of GNR–HA–ALA/Cy7.5-HER2, and its application in HER2/CD44 dual-targeting and fluorescence imaging-guided PDT/PTT combination therapy for breast cancer. Reprinted with permission from ref. 106. Copyright @2019 Elsevier.

In order to develop more effective targeting gold nanocarriers, some researchers consider the introduction of multiple targeting molecules at the same time to enhance the tumor targeting ability and therapeutic effect of gold nanocarriers. Xu *et al.* functionalized HA through Au–S bonds to gold nanorods (GNR), and then coupled 5-aminolevulinic acid (ALA), Cy7.5 and anti-HER2 antibody onto HA moiety for photodynamic therapy (PDT), fluorescence imaging and active targeting, respectively (Fig. 8).^106^ Experiments showed that the dual targeting strategy of HER2 and CD44 could significantly enhance the endocytosis of GNR–HA–ALA/Cy7.5-HER2 in human breast cancer cell MCF-7. *In vivo* studies showed that tumors in mice were completely eliminated without significant side effects.

#### 3.4.2. Silica nanocarriers

Silica nanocarriers mainly include silica nanoparticles, mesoporous silica (MSN), hollow mesoporous silica, and silica nanotubes. Among them, MSNs are porous structures with high drug loading capacity due to large specific surface area and high pore volume.^107^ In addition, the surface of MSNs can be easily modified with functional groups such as amino groups and sulfhydryl groups to further modify targeting molecules, making it a multifunctional and efficient delivery platform for various anticancer drugs. An ideal MSN drug-loading system not only accurately target the tumor site, but also have no drug leakage during blood delivery, and be able to control the release of local high-concentration drugs at the tumor site. Therefore, the surface of MSNs can be modified with gatekeepers that can respond to certain external stimuli (such as pH, oxygen, enzymes, redox reagents, and temperature, *etc.*) to achieve controlled and on-demand drug release.^108^

**Fig. 9:**
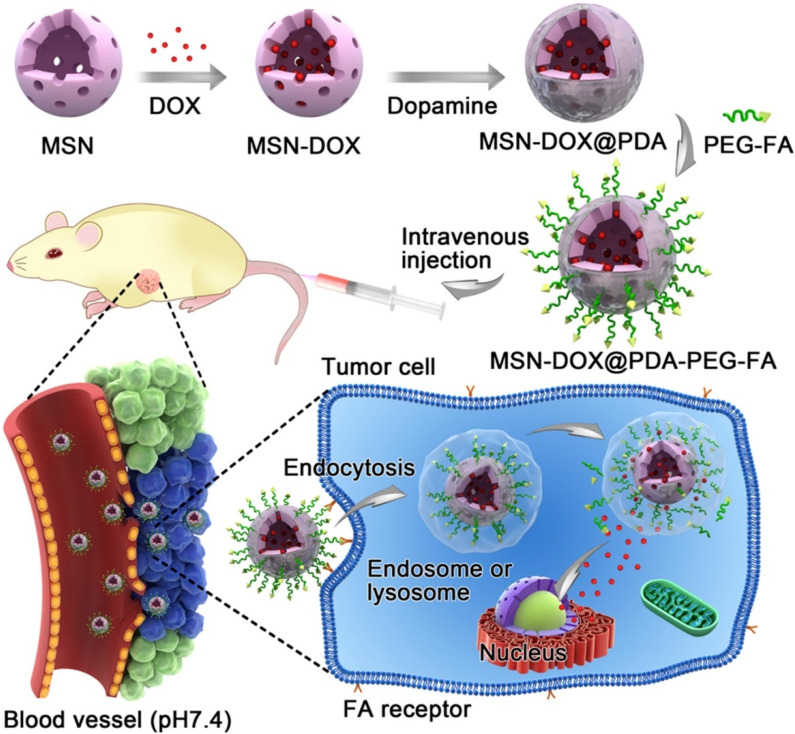
Schematic illustration of DOX-loaded MSN–DOX@PDA–PEG–FA. Reprinted with permission from ref. 109. Copyright @2017 American Chemical Society.

Due to the acidic environment of tumors, which is different from the physiological pH value of 7.4,^108^ this difference can enable the specific release of encapsulated drugs into tumor tissues, and pH-responsive MSNs are becoming more and more popular. Cheng *et al.* coated pH-sensitive polydopamine (PDA) on the surface of mesoporous silica nanoparticles (MSNs), and then introduced FR-targeting molecule PEG–FA to load doxorubicin (DOX) to form drug delivery system (MSNs–DOX@PDA–PEG–FA, Fig. 9).^109^*In vitro* drug release experiments showed that the release of DOX was pH-dependent, reducing the toxic and side effects on normal tissues. They also observed that MSNs–DOX@PDA–PEG–FA had significantly higher targeting efficiency and antitumor efficacy compared with free DOX and DOX-loaded NPs without FA-targeting ligands. Saini and Bandyopadhyaya designed transferrin (Tf)-conjugated, chitosan-coated mesoporous silica nanoparticles (MSNs) loaded with the anticancer drug gemcitabine.^82^ They demonstrated that the nanoparticles release the drug at a very low rate in a neutral environment, but in a large amount in the tumor environment. The Tf-bound MSNs had better uptake on MIA PaCa-2 cells due to Tf–TfR interaction. Most importantly, the modification of Tf increased its killing efficiency against cancer cells to 75%. Lohiya and Katti developed EGFR/HER2 aptamer-conjugated, DOX-loaded, chitosan-coated mesoporous silica nanoparticles (MSNs) for active targeting of EGFR- and HER2-overexpressing breast cancer cells.^83^ The developed targeted MSNs exhibited higher uptake and cytotoxicity against triple-negative and HER2-positive breast cancer cells compared to non-targeted MSNs. Chitosan coating endows MSNs with pH responsiveness and endo/lysosomal escape ability, resulting in precise control of drug release.

**Fig. 10:**
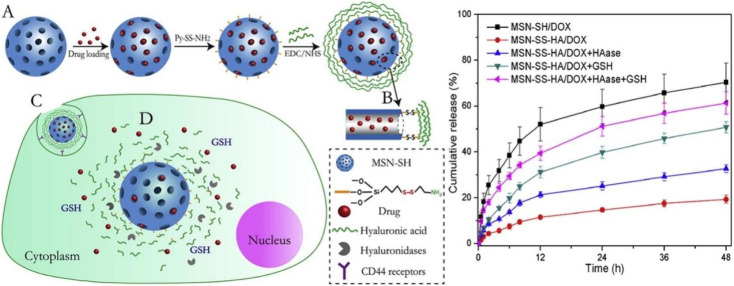
Schematic structure of MSN-SS-HA/DOX drug delivery and cumulative release profiles of MSN-SS-HA/DOX and MSN-SH/DOX in pH 5.0 PBS under different conditions. Reprinted with permission from ref. 112. Copyright @2015 Elsevier.

Glutathione (GSH) is a reducing agent in organisms, and the concentration of GSH in tumor cells is about 100–1000 times higher than that in normal tissues.^110^ This marked difference in GSH levels allows GSH to be used as a selective intracellular stimulant for the development of drug delivery systems that can be degraded by GSH. Disulfide bonds (SS) can maintain stability under normal physiological conditions, blocking the contact of encapsulated drugs with the external environment, but can be reduced by GSH to cause bond breaks and drug release.^111^ Based on this, many researchers have introduced disulfide bonds into nanocarriers to prepare drug-controlled release systems with reduction-sensitive stimuli responsiveness. Zhao *et al.* developed a redox/enzyme dual-stimuli-responsive targeted delivery system.^112^ They grafted HA onto the surface of MSN through a disulfide bond, which not only acts as a gatekeeper, but also can actively target the CD44 receptor on cancer cells. They demonstrated that DOX release was accelerated in the presence of glutathione (GSH) and hyaluronidase (HAase) (Fig. 10). MSN-SS-HA exhibited higher uptake efficiency in HCT-116 cells through CD44 receptor-mediated endocytosis. Venkatesan *et al.* covalently linked the mesoporous shell of mesoporous silica nanoparticles (MSNP) to cancer-targeting Tf *via* disulfide bonds for GSH-controlled DOX drug release in tumor cells.^113^ They demonstrated that Tf on the surface of MSN selectively recognizes TfR on the surface of cancer cells, enabling MSNP-SS-Tf@PEG to be internalized into HT-29 and MCF-7 cells. Then, DOX is released into tumor cells triggered by high GSH concentration.

#### 3.4.3. Magnetic nanocarriers

Magnetic nanoparticles (MNPs) refer to magnetic materials with particle sizes ranging from 0 to 100 nm. Magnetic nanoparticles include hematite, maghemite, nano ferrite, and magnetite.^114^ In particular, iron and iron oxide particles, such as magnetite and its oxidized form maghemite, have unique magnetic properties and biocompatibility, which have been widely used in the fields of drug delivery, tumor hyperthermia, and magnetic resonance imaging (MRI), *etc.* However, due to poor water solubility and easy agglomeration of naked MNPs, plasma proteins are easily adsorbed on the surface and cleared by RES, which poses a certain obstacle to the field of biomedicine. In order to prepare well-dispersed water-soluble MNPs, surface modification and functional group modification are required.

**Fig. 11:**
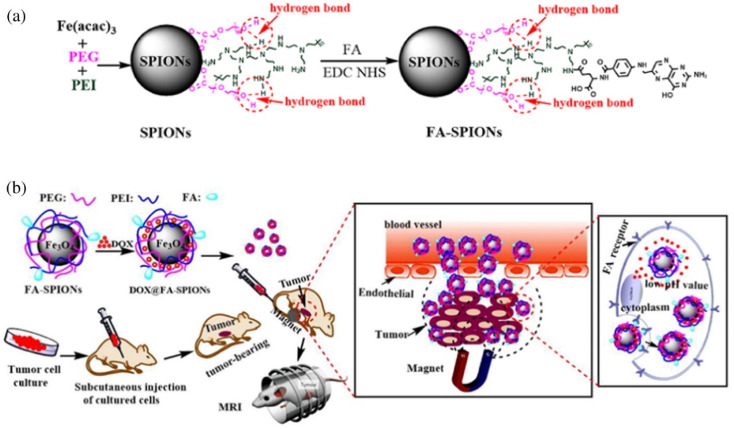
(a) The synthesis and surface coating of SPIONs and FA-SPIONs. (b) The DOX loaded FA-SPIONs for FA-mediated and magnetically targeted drug delivery to the tumor and the MR imaging. Reprinted with permission from ref. 84. Copyright @2017 Elsevier.

The polymer coating adds a protective film to the MNPs, preventing them from being cleared out of the body by the RES due to aggregation. Huang *et al.* designed a nano-delivery system (FA-SPIONs) based on superparamagnetic iron oxides (SPIO) coated with FA-modified polymers (PEI and PEG).^84^ DOX was loaded onto FA-SPIONs through electrostatic adsorption and hydrogen bonding (DOX@FA-SPIONs) (Fig. 11). They found that DOX@FA-SPIONs were actively targeted to MCF-7 cells by FA receptor-mediated endocytosis. DOX@FA-SPIONs achieved MRI monitoring and more effectively inhibited the growth of MCF-7 cells and their xenograft tumors under the action of a magnetic field. Wang *et al.* functionalized the surface of PEGylated SPIO nanoparticles with an anti-EGFR monoclonal antibody (cetuximab) for targeted delivery to EGFR-overexpressing lung cancers.^115^ Compared with non-targeting PEGylated SPIO NPs, anti-EGFR-PEG-SPIO NPs exhibited better targeting ability to H460 tumor cells and enhanced targeted tissue thermogenesis by magnetic resonance-guided focused ultrasound surgery (MRgFUS) therapy. Ghadiri *et al.* developed a dextran spermine-coated iron oxide nanoparticles coupled with Tf to obtain a targeted drug delivery system (TDS-NPs) to specifically target the brain and deliver capecitabine across the blood–brain barrier (BBB).^116^*In vivo* biodistribution and histological studies showed a significant increase in iron concentrations in the brain transported by targeted nanoparticles.

Coating silicon dioxide on the surface of magnetic nanoparticles can obtain magnetic nanocarriers with a core–shell structure. The silica shell can protect MNPs from chemical degradation in the body, avoid the leakage of harmful substances in the nucleus, and reduce the toxic and side effects on normal tissues.^117^ Tabasi *et al.* developed CD44-modified superparamagnetic Fe_3_O_4_/mesoporous silica nanocarriers (NCs-OXA) for the delivery of oxaliplatin (OXA) to HCT-116 colon cancer cells.^118^ They found that OXA was more lethal to HCT-116 cells, which was due to the interaction of CD44 receptors with NH_2_-groups to increase the intracellular uptake of NCs-OXA. Avedian *et al.* designed an iron oxide (Fe_3_O_4_) core coated with mesoporous silica, and then conjugated with FA through PEI to construct a pH-sensitive targeting nanocarrier for loading erlotinib anticancer agent.^119^ The results showed that folic acid-labeled nanoparticles exhibited high toxicity to HeLa cells. Therefore, targeted drug delivery systems can reduce the side effects of anticancer drugs.

Generally, inorganic nanoparticles are more stable and easier in the manufacturing process than liposomes and polymer nanoparticles, which enables them to circulate in the body for a long time. However, it is precisely this point that makes them difficult to degrade in the body and has potential toxicity. Therefore, the research and development of biodegradable inorganic nanoparticles is the direction of inorganic delivery system in the future.

## 4. Conclusion

With the development of medical diagnosis and treatment technology, people's understanding of cancer is deepening, and nanoparticles of various materials have been developed as drug carriers for cancer cell membrane-targeted drug delivery systems. Here, we focus on some recent progress of nanocarriers as an innovative platform for effective delivery and controlled drug release. In order to improve the intratumor delivery and active targeting of drugs, especially for drugs targeting cancer cells, the surface of nanocarriers can be modified with various receptor-targeting ligands (such as, FR, TfR, HA, *etc.*) to specifically bind to the receptors on the surface membrane of cancer cells. Among them, nano-materials containing biocompatible polymers, liposomes or inorganic materials combined with targeting parts show great scope in carrying effective drugs to target sites to enhance therapeutic purposes.

Although the research on targeted nano-delivery system has been invested heavily at present, its clinical application still faces many problems, such as biological distribution, pharmacokinetics, nano-biological interface interaction, and lack of mass production technology. In addition, when used in patients, the potential toxicity of nanoparticles seriously hinders clinical transformation. There is still a lack of accurate *in vitro* and *in vivo* models to evaluate the real patient treatment, which leads to the difference between clinical and preclinical results. Therefore, when designing, optimizing and developing nanoparticles in preclinical stage, there is an urgent need for more evaluation in these aspects. It is worth mentioning that through the joint efforts of scientists and clinicians, more encouraging news can be brought to the active targeting field of anticancer drug nanomaterials in the future.

## Author contributions

The manuscript was written through contributions of all authors. All authors have given approval to the final version of the manuscript.

## Conflicts of interest

There are no conflicts to declare.

## Supplementary Material
